# Seasoning Polymethyl Methacrylate (PMMA) Bone Cements with Incorrect Mix Ratio

**DOI:** 10.3390/ma12193073

**Published:** 2019-09-20

**Authors:** Robert Karpiński, Jakub Szabelski, Jacek Maksymiuk

**Affiliations:** 1 Department of Machine Design and Mechatronics, Faculty of Mechanical Engineering, Lublin University of Technology, Nadbystrzycka 36, 20-618 Lublin, Poland; 2 Section of Biomedical Engineering, Institute of Technological Systems of Information, Faculty of Mechanical Engineering, Lublin University of Technology, Nadbystrzycka 36, 20-618 Lublin, Poland; 3 Orthopaedics Department, Łęczna Hospital, Krasnystawska 52, 21-010 Łęczna, Poland; jacek@maksymiuk.pl

**Keywords:** bone cement, mechanical properties, biodegradation, compressive strength

## Abstract

Cemented joint prostheses are widely used in orthopaedic surgery; however, implants/bone bonds are known to be susceptible to aseptic loosening, particularly in the case of long-term performance. The exact mechanism of this failure is under constant examination. One of the critical factors to the final mechanical functionality of bone cement can be an incorrect mix ratio of a two-component material (powdered polymer and liquid monomer). It can result in the deterioration of the final mechanical strength properties. The paper presents the results from an experimental study on the effects of the deviation from the correct mix ratio on the moisture uptake and the compression strength of cement depending on the seasoning time in Ringer’s solution. The results were subjected to statistical analysis and a mathematical model was developed.

## 1. Introduction

Every year, several million patients require surgical treatment on account of bone defects or to reconstruct joint surfaces resulting from diseases or mechanical injuries. Thus, there is a distinct rise in the number of arthroplasty procedures performed on an annual basis [1,2,3,4]. There are several causes behind the observed tendency, including rising qualifications of surgeons to perform these procedures or the increase in life expectancy. Furthermore, the procedure in question is now being performed in younger patients and there is a wider availability of suitable implants than in the past [5,6]. Joint replacement implants improve the life quality of patients suffering from arthralgia and consequently allow them to function properly. Primary joint replacement releases the patient from pain and inconvenience in a relatively short time.

Advances in implant surgical procedures create a rising demand for innovative implant materials that exhibit the desired physicochemical properties, compatible with human tissues. Among such materials, a large constituent of the group are orthopaedic biomaterials, such as bone cement, which is used as a fixation material in bone surgery and dentistry [7,8].

Acrylate bone cement has been long established as a fixating medium providing a binding interface between bone and prosthetic elements. Cemented joint prostheses are considered to be the “gold standard” in hip and knee prosthetic arthroplasty and have been widely used in orthopaedic surgery since the late 1950s, which was when an endoprosthesis with methyl methacrylate resin was first implanted by Sir John Charnley [9,10,11,12]. Bone cement is extensively used in orthopaedics in arthroplasty applications; however, implants/bone bonds are known to be susceptible to aseptic loosening, particularly in the case of long-term performance. Therefore, there are still certain unresolved problems with bone cement, whose implementation, despite improvements, is burdened with an approximately 5% failure rate within 10 years of implantation, resulting from implant loosening [13,14]. A further issue is osteolysis, a long-term dysfunction of prostheses associated with the accumulation of wear particles at the implant-bone interface as a consequence of the joint’s regular operation; the particles become targeted by the cells of the host immune system–macrophages. As a result, the bone dissolves and significant weakening of the cement–bone connection occurs [15]. The loosening of knee prosthesis implants is a serious problem which necessitates conducting revision surgeries, which in turn reduces the patient’s life comfort and generates substantial costs [16].

There are multiple well-studied causes of implant loosening such as infections, primary malocclusion, or loosening as a result of excessive patient weight. These, however, may be prevented and countered throughout the entire treatment process, which has already led to a significant improvement in the knee replacement procedure results. The outstanding problem is the aseptic loosening of implants, which is the cause of 16% to 42% of revision procedures. Nevertheless, the exact mechanism of aseptic loosening of the prosthesis has not yet been sufficiently understood. It is believed, however, that its occurrence is the combined consequence of material fatigue and chemical degradation resulting from operation in an aggressive environment in vivo [17,18,19].

Due to its complex nature, determining the cause of aseptic loosening of implants is difficult. Factors such as excessive macrophage activation, “stress shielding”, micromotion of implants and high intra-articular fluid pressure have been found to contribute to implant loosening [20,21,22]. 

Bone cement is meant to reduce the risk of prosthesis loosening. In spite of proper cementation techniques having been presented in detail in the relevant scientific literature, unfortunately, only 43% of orthopaedists adhere to all of the recommended steps. The change of mechanical properties of bone cement during the preparation and deposition of prosthesis components may prove to play an important role in the process of aseptic loosening of implants [23,24]. An excessive number of air pores may lead to the formation of microcracks and, as a result, the loosening of an implant. This scenario can be prevented; however, it is critical that the cement is prepared according to the standards. The cement preparation process has undergone gradual improvement with the introduction of vacuum mixing systems, cement application guns or cooling prior to mixing, which extends the polymerisation time [19,25].

Similarly as in the case with most polymer materials, bone cement will also react with the ambient environment, for example by absorbing water, which will act as a plasticiser and change its characteristic properties. Therefore, it must be tested in an environment that most accurately reflects the human body environment, the importance of which increases further given the cement’s long-term viscoelastic properties [23]. Other factors that are critical to the final mechanical functionality of bone cements are error-related, such as incorrect mix ratio or the introduction of contaminants (e.g., blood and tissue residues) into the material structure. Bone cement is a two-component material (powdered polymer and liquid monomer), whose preparation and deposition involves supplementation of various additives that are introduced at subsequent stages, from preparation to implantation. Among contaminants that are frequently introduced into the cement structure during implantation, there are those that are to an extent dependent on the surgeon, such as residual blood or bone tissue. As a result, this may lead to the deterioration of the final mechanical strength properties, or the occurrence of delamination in the assembly [19,26]. 

With respect to the mechanical properties of bone cements, it is vital to examine the impact of aging processes associated with the absorption of physiological fluids and the accompanying hydrolysis of the polymethyl methacrylate (PMMA) that occur in the outermost layers of cement, as well as the effect of deviation from the cement mix ratio recommended by the manufacturer [10,27,28]. Changes in the molecular structure and the plasticising effect of water have been found to reduce the mechanical properties of the tested material. Cement aging is, therefore, considered to be the key factor in the long-term failure in cemented joint replacement [29,30,31].

## 2. Materials and Methods

### 2.1. Material and Sample Preparation

It was decided that the best method to adopt for the investigation of impact of incorrect powder/liquid (P/L) ratios under exposure to Ringer’s solution on the compressive strength of bone cements was to conduct physical tests that involved compressive strength testing of the material under analysis prepared at the recommended and incorrect P/L ratio, understood as insufficient or excessive amount of the liquid component. Although the mix ratio deviations resulting from human operator error typically range between a few to a dozen or so wt %, the extreme imbalance scenarios were included for the sake of statistical analysis. While they are, admittedly, unlikely to occur in clinical practice, the results provided extended data for modelling the tendencies in a greater range of deviations. The adopted range of deviations from the recommended ratio was between –30% and +40% at a variable 10–15% step. The compression tests were carried out on specimens unexposed to Ringer’s solution and subjected to seasoning for 1, 10, 20, and 30 days. In the case of the seasoned specimens, the analysis involved the estimation of the relative weight increase depending on the exposure time. The investigation was performed on DePuy CMW 3 Gentamycin (Raynham, MA, USA) bone cement powder. With respect to dimensions and tolerances, the test samples were prepared in compliance with the procedure described in International Standard ISO 5388:2002, which was further confirmed by the measurements of ready specimens. The cylindrical test specimens were cast in moulds and finished-ground to dimensions (*ϕ*6 mm × ~12 mm). Prior to mixing, the temperature of the powder and the liquid components was lowered to 16 °C, in order to minimally extend the setting time so as to ensure the full penetration of moulds and prevent the premature initiation of cement polymerisation. Limited pre-cooling has been proven not to significantly affect the strength of cement. In surgical practice, the control of cement temperature is performed both prior to its mixing and during polymerisation, which is an exothermic reaction, when the cement is known to reach relatively high temperatures. Although the negative effects of pre-cooling in medical applications are known (the migration of unreacted, toxic monomers to the neighbouring tissues), in the case of extracorporeal, stand tests their medical effects are irrelevant, particularly given that the temperature was decreased by only several Centigrade. 

The compressive strength tests were performed with the use of the MTS Bionix–Servohydraulic Test System (Eden Prairie, MN, USA) for biomedical material testing applications. The other component of the test set-up, the MTS TestWorks software (Eden Prairie, MN, USA), was employed to programme and execute the experiment procedure. The compression speed was specified as per ISO 5388:2002 standard, at 20 mm/min. Despite the referenced standard specifies that 5 specimens should be subjected to testing, the engineering practice determines that in order to obtain more accurate results, it should be 6–7 for each combination of deviation from proportion and seasoning time. For each sample, the force applied to cause fracture was recorded. The obtained results were divided by the original cross-sectional area of the cylinder and, hence, producing their compressive strength. Finally, the results from the calculated average compressive strength of the particular sample batches were subjected to statistical analysis.

The control group, unexposed to Ringer’s solution, was seasoned in the air at 23 °C. The specimens seasoned in Ringer’s solution were weighed after 1, 10, 20, and 30 days of manufacture. Prior to the measurement, all visible residues of the solution were removed from the surface of the samples using a paper towel. The moisture uptake was estimated as the ratio of sample weight gain to the initial weight. The measurements were carried out for an average of approximately 9 samples. The final result is the arithmetic mean of all moisture uptake results within a series.

### 2.2. Statistical Analysis

The statistical analysis set out to determine discrepancies in the compressive strength of specimens produced with incorrect P/L ratio, in the range of deviations from the liquid component content specified in the preceding section, depending on the sample seasoning time in the environment of Ringer’s solution, to which end the Statistica 13.1 package (Tulsa, OK, USA) was employed. The statistical significance level selected for this study was *α* = 0.05.

In order to verify whether the distribution of results from the compressive strength tests of cement specimens formed at incorrect P/L ratios under various seasoning times in Ringer’s solution was close to the normal distribution, three tests were applied: The Kolmogorov–Smirnov, the Lilliefors and the Shapiro–Wilk tests.

The equality of variances was verified with the use of three tests: The F-test (Fisher Test), the Levene’s Test and the Brown–Forsyth Test. In the case with the results that exhibited a normal distribution and an equality of variances, the t-Student test was applied to analyse the differences between the mean compressive strength test results of bone cements formed at incorrect P/L ratios under various seasoning times in Ringer’s solution, at the selected level of significance. The analysis of the results showing the normal distribution and the variance inequality was performed with the use of the Student *t*-test with a separate-variance adjustment (the Cochran–Cox test) for the analysis of the equality of means.

### 2.3. Mathematical Modelling 

In order to fully understand the relation between analysed mechanical properties of the material and the effect of the mix ratio imbalance, the mathematical modelling was applied using Statistica 13.1 software. As the analysis covers a specific, wide range of seasoning times (1, 10, 20, 30 days), the traditional diffusion model was found not to fit correctly. Therefore, a better-fitting linear model, showing high values of the coefficient of determination R^2^, was employed instead. Such modelling allows predicting, to a certain extent, the behaviour of the material in the future. 

## 3. Results and Discussion

Commercially available bone cements are typically sold in ready-to-use sets, which contain components of a precisely measured weight ratio, i.e., a liquid monomer or a loose polymer, often based on polymethyl methacrylate (PMMA) or similar acrylics. The detailed manufacturer’s instructions, supplied with the set, define the mixing conditions (e.g., time and temperature) and methods (e.g., manual, vacuum). Surgical practice indicates, however, that strict adherence to these conditions in situ proves unattainable. At the same time, any deviation from the instructions may result in a change of the mechanical properties of the cement mass, thus affecting the final strength and resistance of the prosthesis-cement-bone connection.

The inaccuracy of the mix ratio in two-component polymeric materials has been known and studied for numerous materials and applications, particularly for adhesives and engineering applications, e.g., [32,33,34,35]. With respect to the optimal mix ratio, the referenced literature sources indicate several scenarios that may occur. In certain cases, it is possible to determine precisely the stoichiometric ratio between the reactants, or the optimal ratio may be expressed as the “at-to range”. For some materials, however, even strict observance of the stoichiometric equivalence does not ensure that the cured material will exhibit the optimal strength properties. A prominent example concerns the ratio balance between a curing agent and an epoxy resin, which if retained, promotes the strength and heat resistance of the material; however, the contradiction that occurs is that while the former benefits from low cross-link density, the latter is the opposite. Furthermore, certain materials when formed at the imbalance in favour of the curing agent, compared to the stoichiometric ratio, become more flexible or more brittle when, in fact, formed at insufficient hardener content. On the other hand, the hardener-rich polymer material will demonstrate higher tensile/compression strength and greater heat and chemical resistance. Engineers may consciously modify the proportions (although typically in a narrow range), nevertheless, precise performance tests are necessary to determine which concentration is optimal for which application. The optimised ratio should then be tested under actual operating conditions, in case there were other variables that could potentially affect the material’s performance (i.e., joint weight and geometry, curing conditions, a combination of environmental conditions) [34]. A critical factor determining the long-term performance of cements, i.e., firm stability of hip replaced in arthroplasty, is its resistance to the effects of the aggressive in vivo environment. What this includes is the aging of the cement material in the presence of body fluids, which may, in turn, lead to joint damage, necessitating revision procedures or be the source of discomfort to the patient and financial strain. Experimental aging of cement in Ringer’s solution (an isotonic aqueous solution of several salts, relative to human body fluids) may, therefore, provide essential insights into the processes that occur during aging in vivo, as well as highlight potential sources of subsequent defects. Three processes are known to occur during bone cement aging: Cement polymerisation over extended time periods, the material and joint strength increase, diffusion of unreacted cement, and liquid penetration into the cement mass [10,36]. It should be remembered that PMMA cements exhibit highly hydrophobic properties [37].

**Figure 1:**
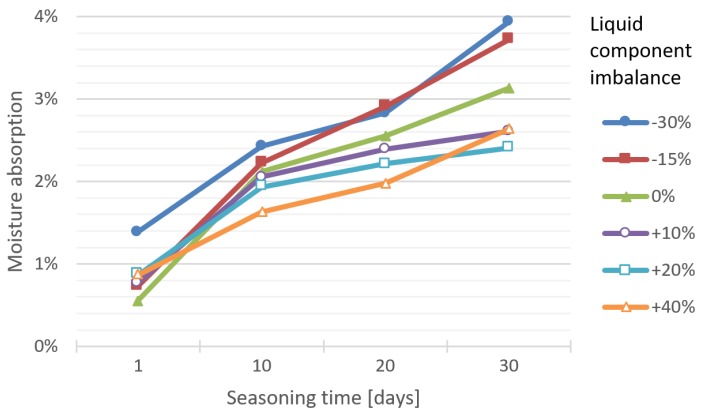
Dependence between moisture absorption and seasoning time in Ringer’s solution of cement specimens prepared at different P/L ratio imbalances.

Figure 1 shows the results of liquid absorption test of bone cement specimens depending on the seasoning time in Ringer’s solution and accounting for the P/L ratio imbalance.

With a view to providing a better understanding of the observed processes, the subsequent stage of the analytical works consisted in the adjustment of the developed mathematical model in order to reflect the dynamics of occurring changes and to describe the global behaviour of the cement material, depending on the parameters in question. The statistical works focused on a linear model, which exhibits good adaptability to the results from the experimental tests for the analysed range. While the scientific literature in the field tends to prefer the exponential model as the most suitable for the description of the studied phenomenon, it did not provide an accurate description in the case of our data. The exponential model provides satisfactory accuracy concerning the results obtained over shorter seasoning times than it was performed here. The mathematical modelling, performed as part of the study, showed that the initial diffusion of water into the cement during seasoning in Ringer’s solution (up to 72 h) follows the Fickian diffusion model [10]. Given the wider time horizon that this study was performed in—over the period of 30 days, it was the linear model (Table 1) that showed a better fit (R^2^ = 0.79/0.98) with the results from experimental tests than the exponential model, hence the former was selected for this study.

In most test series, the accuracy of the linear model, expressed with the coefficient of determination R², is very good (>0.9), in two series good (0.8–0.9), or borderline good to satisfactory (0.6–0.8). The course of linear regression clearly displays a close relationship with the liquid component imbalance in the cement mix ratio: The smaller the amount of liquid, the more solution the specimen absorbs. The directional coefficient (slope) decreases (and so does the absorption of the fluid) with the increase of the proportion of the liquid part—the dilution of the cement.

The specimens deficient in the liquid component exhibited a notably greater Ringer’s solution absorption tendency at any point during the seasoning. A further interesting observation is that, regardless of the amount of liquid component excess, upon a 30-day exposure, the degree of absorbability is practically equal, whereas in specimens of shorter exposure time it has been observed to depend directly on the liquid component excess level. On exposure day 10 and 20, the dependence between the excess of the liquid component and liquid absorption becomes evident, i.e., the larger the share of the liquid component in the total mix ratio, the lower the absorbability of the bone cement material.

The observed behaviour of the material may be to a certain extent explained by the initial diffusion of the monomer, leading to the formation of cavities in the cement, which are subsequently filled by water molecules, hence increasing moisture absorption over the initial 72 h of exposure (the Fickian diffusion model). The process decelerates over a longer period of time due to a decrease in the amount of the diffused monomer. The moisture absorption time span is known to be regulated by molecular changes in the cement and the relaxation of polymer chains. What is more, cement porosity can prolong the moisture absorption process, by creating paths for further diffusion [10]. 

**Figure 2:**
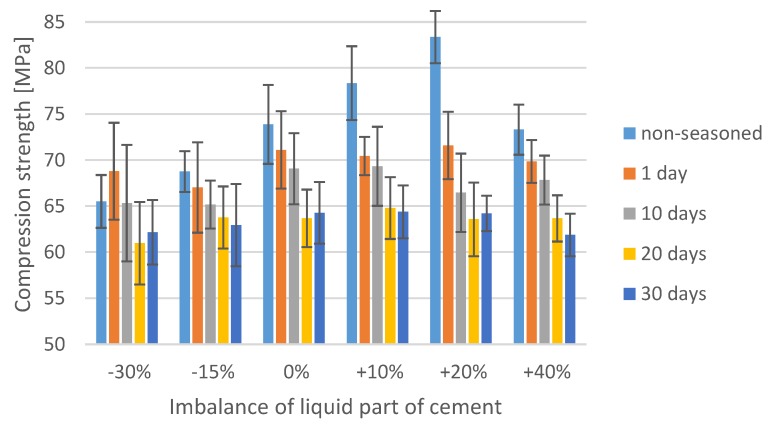
Compression strength after seasoning in Ringer’s solution of samples with an unbalanced mix ratio.

The explicitly negative nature of porosity in the cement material is contested by certain researchers [38], who indicate that the poriferous cement provides a surface advantageous from the perspective of adhesive processes. Its surface is understood to boost cement adhesion to bone and prosthesis, e.g., by micro-anchoring the bone tissue in porosities and bone penetration into the cement. In addition, surface preparation methods for adhesive bonding in engineering applications almost exclusively aim to increase the surface roughness, which ultimately leads to the development of stronger and more durable adhesive bonds [39]. The water inside the cement material could act as a plasticiser, causing structural changes and degradation of the polymer structure, as described by other authors [40,41]. Although cement initially ensures the mechanical stability of implants, in prolonged use, it is reported to reduce in strength. In extreme scenarios, cement loosening or cracking may occur. Aging has been observed to induce changes in the mechanical properties of bone cements, and it has been established that prolonged aging in Ringer’s solution reduces the compressive mechanical strength of the implant material (Figure 2).

**Figure 3:**
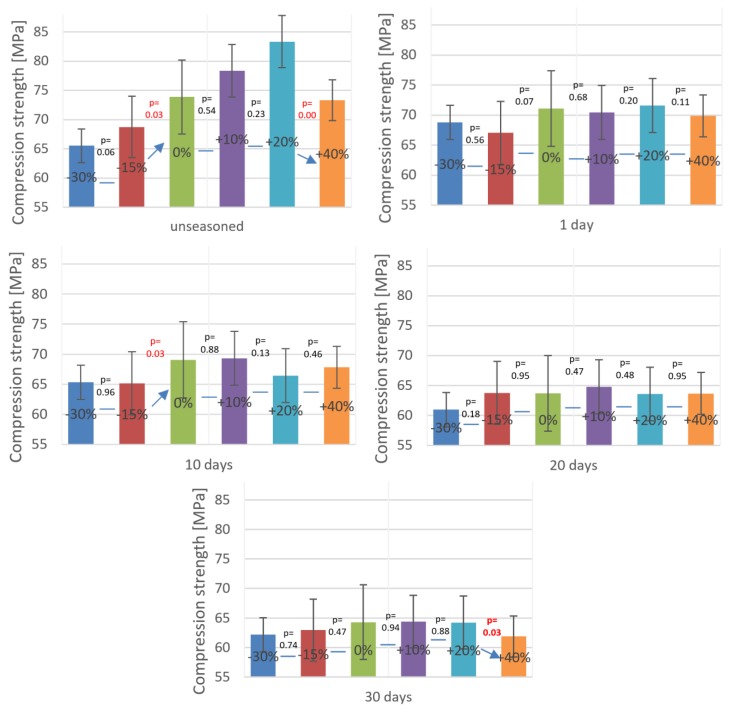
Compression strength after seasoning in Ringer’s solution of samples with an unbalanced mix ratio.

In order to thoroughly analyse the interdependence between the cement mass ratio deviation with the effect of seasoning in Ringer’s solution on the mechanical strength of the test material, a statistical analysis of the results obtained from experimental tests was performed. The software package used to analyse the data was Statistica, which allowed us to attempt to assess the impact of the factors in question (deviations from the recommended P/L ratio and Ringer’s solution exposure time) on the final compressive strength of cement specimens. To fulfil the prerequisites for the application of the ANOVA method, the data obtained from the strength tests were analysed to verify the normality of distribution and homogeneity of variances. The conducted analyses confirmed that the distribution of results is normal, however, their variance was found to be heterogeneous. Therefore, as previously stated, in the absence of homogeneity of variances the classic statistical Student *t*-test was extended to account for separate estimates (the Cochran–Cox test). The results from the statistical analysis presented in Figure 3 and Table 2 have been grouped according to specific bone cements P/L ratios. The horizontal lines indicate the results from the strength tests of a series of specimens for which the statistical analysis did not show a statistically significant difference in means.

In addition, an equality test of paired samples was carried out for compressive strength of cement specimens formed at incorrect P/L ratios under various seasoning times in Ringer’s solution. In Table 2 below, red colour denotes the results whose statistical analysis indicates the inequality of means. The average differences between the compared strength values are given in brackets.

One of the key findings emerging from the statistical analysis part of the study is that the average compressive strength of bone cements upon exposure in Ringer’s solution is less than 70 MPa, which is the value specified as the minimum compressive strength of bone cement in the International Standard ISO 5833. The general tendency of bone cement specimens exposed to 10 days of seasoning in the solution, regardless of the deviation from the cement mass ratio, is that their average strength fell below the recommended threshold. In addition, on day 20 and 30 of seasoning, only in several samples, and only among the ones of correct P/L ratio, the strength reached the minimum 70 MPa. However, the main limitation of our study that ought to be noted is that the samples were aged and tested at room temperature (20–22 °C), while their actual operating conditions are much more severe: Upon fixing, the prosthesis bone cement is exposed to regular human body temperature (36.5–37.5 °C). Hence, it could be hypothesised that subjecting the identical sample material to tests at elevated temperature would result in different, presumably lower compressive strength values. As remarked by other researchers [10], the ISO 5833 standard fails to clearly specify the clinical effectiveness of bone cements, focusing on the initial state of bone cement material, and the effects of physiological aging not being addressed. The use of Ringer’s solution in this study, however, is to a certain extent also a limitation: This approach does not fully recreate the precise conditions inside the human body. Nevertheless, it does provide aggressive environment conditions in vivo, providing the basis for drawing valid conclusions regarding the dynamics of cement strength changes. It should also be emphasised that the cement used for the prosthesis implantation is practically from the beginning subjected to variable stresses associated with immediate patient verticalisation. Therefore, only long-term material fatigue testing is capable of providing a precise answer to questions related to cement strength. Finally, the cement mass preparation was done by manual mixing technique in accordance with ISO 5833, although vacuum mixing devices are sometimes used clinically. Vacuum mixing of bone cement is likely to affect the porosity and thus the obtained results [38].

Although these were the strength parameters of cement material that the presented study was focused on, its adhesive properties are of equally high relevance for surgical applications. Any modification of the cement composition can, as demonstrated, degrade the static strength of bone cement, and negatively affect its adhesive properties, similarly as in the case with other polymer-based materials [42,43]. 

**Figure 4:**
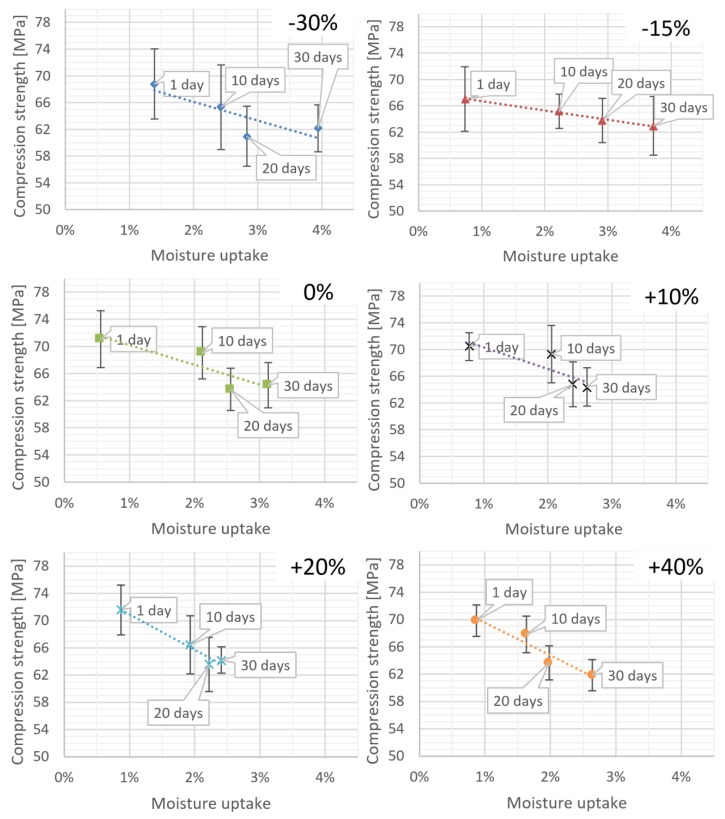
Specimen weight increase (moisture uptake) and corresponding compressive strength depending on seasoning time in Ringer’s solution.

As part of statistical investigations, the strength characteristics of bone cement specimens were analysed. The mean compressive strength was collated with the absorption values of the cement specimens prepared at various P/L ratios. As expected, the results indicate that the material strength increases along with an increase in Ringer’s solution uptake; what is more, the character of these changes shows a clear dependence on the P/L ratio in the cement mix. Figure 4 presents the overall summary and the corresponding linear models.

From the analysis of the corresponding linear models, it can be seen that the increase in the liquid component in the cement mix results in the drop in the compressive strength of the specimens. It was found that upon exposure to Ringer’s solution, the cements with a greater amount of the liquid monomer show a higher decrease in the compressive strength compared to “dry” specimens, with low monomer liquid content. This is further confirmed by the decreasing slope of the regression line for the specimen models arranged in an increasing order of L component in the cement mix (Table 3).

The changes observed with the increasing content of the liquid component resemble the former linear absorption model, i.e., there is a marked increase in the absolute value of the slope coefficient of the model.

The results above have been compiled into a surface plot (Figure 4), which collectively shows the interrelations between the three parameters under scrutiny, i.e. the exposure time of specimens to Ringer’s solution, the cement mass mix ratio imbalance, and the resulting average compressive strength of the cement material. The estimation of the non-linear model takes the following Equation (1):

(1) $$Compression\;strengt=b_{1}+b_{2}\times v_{1}+b_{3}\times v_{2}+b_{4}\times v_{1}{}^{2}+b_{5}\times v_{1}\times v_{2}+b_{6}\times v_{2}{}^{2}$$

where: $v_{1}$—excess/deficiency of liquid part in the composition of cement in %, $v_{2}$—seasoning time in Ringer’s solution in days.

**Figure 5:**
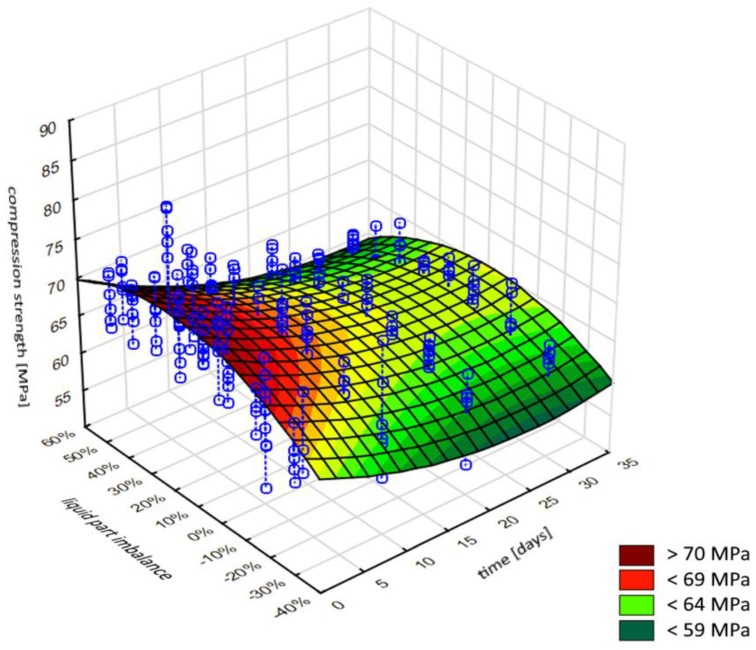
Model of relations between mix ratio imbalance and seasoning time in Ringer’s solution to the average compressive strength of bone cement.

The result of the analysis is the model shown in Figure 5:

The parameters of the obtained model (coefficients) are as follows:
b_1_*b*_2_*b*_3_*b*_4_*b*_5_*b*_6_72.31−0.589.550.01−0.15−23.00

The calculations have shown that five of the parameters are statistically significant, and only the $b_{5}$ coefficient (*p* = 0.09) cannot be treated as statistically significant. Therefore, the analysis was repeated with the omission of the component $b_{5}\times v_{2}^{2}$ (the product of seasoning time and mix ratio imbalance).

The final statistically significant model coefficients were obtained, see Equation (2):

(2) $$Compression\;strength=b_{1}+b_{2}\times v_{1}+b_{3}\times v_{2}+b_{4}\times v_{1}^{2}+b_{5}\times v_{2}^{2}$$

*b*_1_*b*_2_*b*_3_*b*_4_*b*_5_72.54−0.597.650.01−23.41

The R coefficient describing the accuracy of the model has amounted to R = 0.69, which indicates a satisfactory fit level of the adopted model with respect to the experimental data.

## 4. Conclusions

From the study results reported in this paper, it emerged that bone cements produced at “various” P/L ratio imbalances from the manufacturer-recommended composition that are not subjected to seasoning exhibit statistically significant difference in the compressive strength. Therefore, it is essential that the methods for the bone cement preparation during arthroplasty are closely and meticulously followed, and that the process is executed with utmost precision. This strict adherence is dictated by the high sensitivity of the cement material to even the smallest discrepancies in the P/L mix ratio. Over time, a slight deficiency of the liquid component will increase the liquid absorption rate by the cement, effectively leading to the loosening of the implants.

Currently, no contraindications exist against the full loading of cement-fixed implants immediately upon surgery. However, the results from our study show that over time the discrepancy between the strength of badly and well-prepared cement diminishes. Bearing this dynamics in mind, it should be recommended that the limb loading in the early postoperative period ought to be restricted, otherwise the risk of aseptic loosening of the implant increases.

As proven, the absorbed liquid exerts a negative impact on the physical properties of the cement, what is more, it may come into a chemical reaction with each component, which requires further investigation but, on the other hand, more importantly, makes a case for the extension of the diagnostic works particularly to emphasise testing in the environment reflecting the human body conditions (temperature 36.6 °C, intra-articular pressure).

The effect of Ringer’s solution on the properties of cement demonstrated in our study carries serious implications for research into increasing the resistance of bone cement to the impact of such factors.

## Figures and Tables

**Table 1:** Goodness of fit of the linear model of cement moisture uptake depending on the material preparation strategy.

P/L Ratio Imbalance (Liquid Component)	Parameters of the Linear Model of Moisture Uptake (mx + b)		
	m	b	R^2^
−30%	0.0081	0.0063	0.97
−15%	0.0088	0.0013	0.94
0%	0.0065	0.0031	0.79
+20%	0.0051	0.0059	0.88
+40%	0.0057	0.0037	0.98

**Table 2:** Results from statistical analysis of liquid component imbalance vs. equality of mean strength values.

**non-seasoned**									**1 day seasoned**							
Liquid component imbalance		−30%	−15%	0%	+10%	+20%	+40%		Liquid component imbalance		−30%	−15%	0%	+10%	+20%	+40%
vs. seasoned:	**non-seasoned**	-	-	-	-	-	-		vs. seasoned:	**non-seasoned**	=	=	≠	≠	≠	≠
	1 day	=	=	≠	≠	≠	≠			1 day	-	-	-	-	-	-
	10 days	=	≠	=	≠	≠	≠			10 days	=	=	≠	=	≠	=
	20 days	=	≠	≠	≠	≠	≠			20 days	=	=	≠	≠	≠	≠
	30 days	=	=	≠	≠	≠	≠			30 days	≠	≠	≠	≠	≠	≠

**10 day seasoned**									**20 day seasoned**							
Liquid component imbalance		−30%	−15%	0%	+10%	+20%	+40%		Liquid component imbalance		−30%	−15%	0%	+10%	+20%	+40%
vs. seasoned:	**non-seasoned**	=	≠	=	≠	≠	≠		vs. seasoned:	**non-seasoned**	=	≠	≠	≠	≠	≠
	1 day	=	≠	=	=	≠	=			1 day	=	=	≠	≠	≠	≠
	10 days	-	-	-	-	-	-			10 days	=	=	=	≠	≠	≠
	20 days	=	=	=	≠	≠	≠			20 days	-	-	-	-	-	-
	30 days	=	=	≠	=	≠	≠			30 days	=	=	=	=	=	=

**30 day seasoned**																
Liquid component imbalance		−30%	−15%	0%	+10%	+20%	+40%									
vs. seasoned:	**non-seasoned**	=	=	≠	≠	≠	≠									
	1 day	≠	≠	≠	≠	≠	≠									
	10 days	=	=	≠	=	≠	≠									
	20 days	=	=	=	=	=	=									
	30 days	-	-	-	-	-	-									

**Table 3:** The linear model of moisture uptake depending on seasoning time in Ringer’s solution.

Mix Ratio Imbalance (Liquid Component)	Parameters of the Linear Model of Compressive Strength (mx + b)		
	m	b	R^2^
−30%	−277.3	71.7	0.69
−15%	−140.3	68.1	0.99
0%	−290.4	73.1	0.78
10%	−321.1	73.5	0.73
20%	−519.2	76.1	0.96
40%	−478.8	74.3	0.92
